# The Network That Unites a Qualitative Study on Clinical Psychological Intervention for Women with a History of Breast Cancer and Chronic Pain

**DOI:** 10.3390/ejihpe12060046

**Published:** 2022-06-11

**Authors:** Marilena Maglia, Julie Tortorici, Vittorio Lenzo, Daniela Aiello, Marco Alì, Rosanna Aiello, Pasquale Caponnetto

**Affiliations:** 1 Department of Education, University of Catania, 2 Ofelia, 95124 Catania, Italy; m.maglia@unict.it (M.M.); julie.tortorici@studium.unict.it (J.T.); pcapon@unict.it (P.C.); 2 Center of Excellence for the Acceleration of HArm Reduction (COEHAR), Department of Clinical and Experimental Medicine, University of Catania, 95131 Catania, Italy; 3 Department of Social and Educational Sciences of the Mediterranean Area, University for Foreigners “Dante Alighieri” of Reggio Calabria, 89125 Reggio Calabria, Italy; 4 Istituto Clinico Catanese Humanitas, SP54 Contrada Cubba M., 95045 Misterbianco, Italy; daniela.aiello@ccocatania.it (D.A.); marco.ali@ccocatania.it (M.A.); rosanna.aiello@ccocatania.it (R.A.)

**Keywords:** psycho-oncology, chronic pain, psychological support, qualitative study, treatment

## Abstract

The aim of this qualitative research is to deepen the knowledge in the field of psycho-oncology and the consequences of chronic and persistent pain by listening to patients’ experiences, their emotions and difficulties in facing this hard condition, and assessing their perception of the role of the psychologist in pain management. In this qualitative study, a semistructured interview was used, designed from three research questions: chronic pain and quality of life; chronic pain and psychological well-being; and the role and perception of the psychologist in pain management. The sample consists of 29 women who suffered or have recovered from breast carcinoma, and who currently report having chronic pain due either to the presence of the cancer or as a result of surgery or treatment. Three themes emerged from the thematic analysis: quality of life and psychological well-being, relational well-being, and perception and role of the psychologist. Two subthemes have been identified for each theme: common features of chronic pain and consequences and resilience for the first theme; not feeling understood and willingness to protect loved ones for the second theme; and improvements perceived by users and reasons for not making use of the service for the last theme. In conclusion, the results obtained from the literature and those from the analysis of the interviews are discussed and compared, and reflections are made on possible future implications.

## 1. Introduction

The International Association for the Study of Pain (IASP) defines pain as: “An unpleasant sensory and emotional experience associated with, or resembling that associated with, actual or potential tissue damage, or described as such” [1]. Pain is one of the most important problems, both because of its high prevalence (50–60% in every phase of the disease, up to 70–85% in advanced stages) and because of its serious repercussions for the patient (worsening quality of life, increased risk of developing psychopathological disorders, particularly depression, and risk of suicide) [2]. Pain has a multifactorial pathogenesis, consisting of organic factors (e.g., injury), cognitive factors (expectation and meaning attributed to pain), and emotional factors (stress, anxiety, and depression). In cancer diseases, pain may be present at diagnosis, during or after treatments, and in advanced stages [1]. The expression of cancer pain is the result of many different components such as the perception of physical pain, psychological, social, and spiritual suffering, the patient’s beliefs, and the influence of the external environment [3]. In particular, chronic pain persists beyond the duration of tissue damage or healing of such damage. Location and intensity vary over time and fluctuations in the latter are common. It may be accompanied by changes in personality, lifestyle, functional abilities, and depressive signs and symptoms due to the fact that it may lead to immobility, dependence on others, difficulty in resting at night, reduction in appetite, and complications in the continuation of treatment [3]. 

According to the AIRC (Italian Association for Cancer Research), depending on the cause, chronic cancer pain can be felt as: pinpricks, tingling, painful cold sensations or other forms of altered sensation, burning or shaking, and cramping or stabbing, and it can be deep, dull or throbbing. It may be a consequence of either the presence of the tumour or the surgery or treatments. 

Because of its characteristics, chronic pain has a strong impact on quality of life [4] and, especially if it is not controlled and treated, can lead to numerous psychological and social problems such as sleep and appetite disorders, reduced physical activities, possible negative effects caused by excessive medication, loss of self-esteem, anger, depression, and anxiety [4]. Numerous studies have focused particularly on the latter two disorders, since they are frequently observed in patients suffering from chronic pain [5]; major depressive disorder is present in 50% of cases and anxiety disorder in 60%, but this varies according to the type of pain (a lower rate, between 1% and 27% for neuropathic pain, and a higher rate, between 18% and 60% for fibromyalgia pain) [5]. 

Depressive disorder in chronic pain patients is very often underestimated and undiagnosed, and consequently not treated properly [6]. It is interesting to point out the interdependence between chronic pain and depression, which has immediately interested many scholars, so frequently that it has been labelled “depression–pain syndrome” or “depression–pain dyad” [7]. 

Pain is not only associated with major depressive disorder but also with anxiety disorders, especially post-traumatic stress disorder, panic disorder, and social anxiety disorder [8]. Anxiolytics, such as antidepressants, are also beneficial for both anxiety disorder and reduction in pain intensity; furthermore, neuronal circuits involved in anxiety have been analysed which, when moderately activated, lead to a state of hyperalgesia [8]. It is precisely the characteristic feature of chronic pain, its persistence, that causes worry, agitation, and gives rise to a state of anxiety [9]. 

An interesting aspect is the relationship between chronic pain in oncology and relational well-being. “Social withdrawal is defined as keeping away, withdrawing from social opportunities” [10]; this phenomenon is often present in patients suffering from chronic pain and the causes may be numerous. First of all, the feeling of not being understood by relatives or friends prevails because of embarrassment and guilt linked to the condition, which leads to reduced social contacts [11]. Those suffering from chronic pain are physically debilitated and tend to avoid any kind of activity and behaviour that might increase the perception of pain; these restrictions force the patient to deny themselves moments of leisure that they might also share with family and friends [9]. Research, in general, has underlined the growing need for health professionals to work alongside psychologists in order to implement treatments that could manage the presence of pain in all its facets (in addition to medical, there are also cognitive, affective, and social ones) [12]. 

It is now clear that the psychological components in the pathogenesis and symptomatology of chronic pain are just as influential as the medical ones, and that psychosocial aspects significantly affect the patient’s quality of life and perception of the disease. It is worth noting a burgeoning and recent literature has zeroed in on the central role of psychological functioning in the context of several medical chronic conditions including cancer [13]. In addition to improving the understanding of variance in anxiety, depression, and quality of life levels, knowledge on the underlying psychological factors would have clinical implications for psychological treatment. A breast cancer diagnosis is assumed to be a traumatic event that women may face during their lives and is characterized by chronic stress [14,15,16]. Regarding its psychological impact, a recent literature review has well-depicted the complexity of the consequences of breast cancer, especially for women under fifty [17]. Precisely for these reasons, qualitative methodology represents an essential tool for disentangling in depth the effect of breast cancer among people. Though this kind of research struggles with some limitations, it provides a conceptually worthwhile framework for implementing psychological intervention.

In this light, it is preferable to use a multidimensional and interdisciplinary approach, within which a psychological treatment, combined with medical therapies, is of fundamental importance [18]. Psychological intervention approaches can be divided into four categories: self-regulation, behavioural, cognitive–behavioural, and acceptance and commitment therapy [18]. 

The first aims to manipulate psychosocial variables (behaviour, motivation, and cognition) to modify the experience of pain. Self-regulation intervention uses the mind–body connection to try to increase the capacity for self-control over emotional and physiological factors, which tend to be difficult to manage consciously [18]. 

Behavioural therapy, which is based on operant conditioning strategies, treats all pain-related behaviours such as excessive verbalisation of suffering, facial expressions, restriction of movements, and frequent discussions on the subject. It aims to reduce these behaviours and reinforce positive ones by involving not only the person concerned but also close friends and family members, who very often unconsciously reinforce wrong behaviours [18]. 

Cognitive–behavioural therapy, the most widely used in the treatment of this condition, works by reducing highly anxious thinking about pain and future pain [19]. This type of treatment aims in particular at modifying maladaptive emotions, behaviours, and cognitions through a systematic goal-oriented procedure [18].

Finally, acceptance and commitment therapy, which promotes the achievement of acceptance of pain (consisting of the willingness to experience pain and commitment to life’s previous activities despite the suffering condition), can have good results, focusing the individual’s efforts and energies no longer on trying to avoid unavoidable pain but on improving their life [18].

Since the results obtained with all these types of strategies are not obtained with medical therapies, it is essential not to underestimate the role of the psychologist in the whole management of chronic pain.

Based on these premises, this study aimed to investigate through a qualitative approach the experience of chronic pain among women with a history of breast cancer. Specifically, we sought to explore in depth the patient’s emotions and difficulties while struggling with cancer as well as their perception of the role of the psychologist in pain therapy.

## 2. Materials and Methods

### 2.1. Research Questions

The research questions of this qualitative study address the following thematic areas:

- Chronic pain and interference with quality of life and psychological well-being (How does your physical pain interfere with daily activities? How does your physical pain interfere with your psychological well-being? Tell me about what you feel, how you feel.);
- Chronic pain and relational well-being (Does physical pain also interfere with your relationship with family members or friends? In what ways? How do you feel when you are able to vent to your relatives or friends about his/her mental or physical suffering?);
- Participants’ perception of the role of the psychologist and the usefulness of psychological support in the management of chronic pain. (Have you ever sought help from a psychologist for this physical and/or psychological suffering of yours? Was it helpful to you? Tell me about your experience; if you have never done so, do you think it would be helpful to you and why? Have your family members ever used psychological support for their emotional state related to your neoplasm? Was it helpful for them and for coping better? Please tell me about their experience; if they have never used it, do you think it can also be helpful in supporting you in your treatment journey? Justify your answer.).

### 2.2. Participants

The qualitative research was carried out with a sample of 29 women, who suffer or have recovered from breast carcinoma, and who currently report having chronic pain due either to the presence of cancer or as a result of surgery or treatment. The sample initially consisted of 32 participants, but three were excluded since they marked 0 on the Verbal Rating Scale (VRS) of pain at the time of the interview.

### 2.3. Methodology

The study was approved by the Ethics Committee of the Department of Educational Sciences of the University of Catania (n. 2021.07.19/3). All procedures were conducted in agreement with the ethical norms set by the Italian National Psychological Association. In this study, the qualitative approach was used to gain in-depth knowledge of cancer patients’ experiences, explore their experiences, and understand to what extent psychological support is sought in situations of physical suffering and how helpful it can be in promoting mental well-being. The present experimental research followed the “SRQR” standards and guidelines of qualitative research [20]. Data were collected by means of a semistructured interview, the creation of which was characterised by review and control phases. 

The interview is initially made up of general questions that provide us with the following information:

- Name, age, marital status, and any children;
- Duration of therapy;
- Type of treatment followed;
- “Verbal Rating Scale” for chronic pain, a scale to rank perceived severity from 0 to 3.

In the second part of the interview, there are 8 open questions that originate from the 3 research questions previously mentioned and explore the following thematic areas:

- Physical pain and interference with daily activities;
- Physical pain and psychological well-being;
- Physical pain and interference in relationships with family or friends;
- Ability to vent to family or friends;
- Any requests for psychological support because of physical or psychological suffering related to the disease, experience, and perception of the psychologist’s role;
- Possible request for psychological support from family members for their emotional state related to the presence of the patient’s illness, experience, and perceived usefulness of the patient.

### 2.4. Eligibility Criteria

Participants were eligible for the study if:

1. They are female;
2. They suffer or have recovered from breast carcinoma;
3. They currently claim to have chronic cancer pain, assessed by the Verbal Rating Scale.

### 2.5. Recruitment and Sampling Strategy

The study was conducted at the Istituto Clinico Catanese Humanitas, Misterbianco, Catania. The study was implemented by the external research team with the contribution of the hospital staff. The participants who took part in this research were identified from medical files and according to the eligibility criteria. During the recruitment period, all the women who were eligible were invited to participate to this study. All persons invited were explained the purpose and the procedures of the research. Women’s participation in the study was completely voluntary.

The nonprobabilistic sampling technique, often preferred in qualitative research, was used. This type of strategy allows for the selection of participants while respecting the research objectives and eligibility criteria. Quota sampling [21] was used to predetermine the choice of the number of participants, which equals 29 since according to J. W. Creswell more than 25 individuals are needed for qualitative research [22].

### 2.6. Data Collection and Processing

The interview was administered by telephone, using the paper and pencil methodology. The interviewers were part of the research team to avoid possible influences on the interview process. The women unanimously agreed to audio-record the entire duration of the administration, which was about thirty to forty minutes for each of them. An isolated location free of acoustic interference and other interruptions was chosen for the administration of the interview. Each participant was provided with an informed consent form, which was to be signed and returned, where the objectives and aims of the research and the rights of each person in line with the ethics of psychological research are explained and anonymity, confidentiality, and waiver are guaranteed.

### 2.7. Data Management and Analysis

All text interviews were transcribed within a week of each interview and Microsoft Word 2016 was used for formatting. In this study, thematic analysis was used, which is a “method for identifying, analysing and reporting patterns (themes) within data” [23]. This tool allows us to identify common patterns and characteristics that emerged during the interviews [24] regarding chronic cancer pain, giving us a detailed and in-depth account of the results obtained and allowing themes to emerge, which are the main product of the data analysis [23]. This, as pointed out by V. Braun and V. Clarke, is carried out following six steps, which were followed in detail in this research. The first is characterised by repeated in-depth readings of the interview transcripts in order to study them carefully; each text is memorised by identifying salient information and drawing up summaries. Subsequently, coding systems are developed, which include both deductive and inductive codes; the former is derived from the research objectives, the latter from what emerged from the analysis of the interviews. Codes allow the analysis of data to be directed by selecting, from a large amount of information, what is most relevant, and organising the text around a common description. Such codes, once applied to all transcripts, are reorganised into themes, defined as “a pattern found in the information that describes and organises observations and interprets aspects of the phenomenon [25]”. The themes that emerged, with their respective subthemes, result from the three research questions. Each theme is described and illustrated with reference to the transcripts and using literal quotations from the participants as explanatory models.

## 3. Results

This section presents in the first place the characteristics of the participants, providing then a general overview of the issues raised, followed by a more detailed description.

### 3.1. Characteristics of Participants

All characteristics with the corresponding percentages are summarised in Table 1. Twenty-seven women out of twenty-nine (93.10%) are married and two are (6.89%) divorced; among these, twenty-seven (93.10%) have children and two (6.89%) do not.

During the interviews, the Verbal Rating Scale (VRS) was also administered, a short scale to identify the level of severity of perceived pain. Out of twenty-nine women, seven (24.13%) indicated level 1, eighteen (62.06%) level 2, and four (13.79%) level 3. Fifteen participants (51.72%) received psychological support and fourteen (48,27%) did not.

### 3.2. Themes

Three themes emerged from the thematic analysis, with their respective subthemes listed in Table 2.

#### 3.2.1. Theme 1: Quality of Life and Psychological Well-Being

Careful and repeated re-reading of the interviews revealed that chronic cancer pain greatly influences both the daily activities and the psychological well-being of the participants. In particular, a positive correlation emerged between higher VRS values (2–3) and the greater difficulties experienced by women in these two related areas. However, in spite of the many negative consequences expressed by women in both psychological well-being and daily activities, which are listed shortly, a common resilience reaction was found.

Subtheme 1: Common Features of Chronic Pain and Consequences

The chronic pain experienced by the sample is often a consequence of either hormone therapy, chemotherapy or surgery. Most women complain of problems with their joints, bones, back, feet, hands, and legs, especially when they wake up in the morning. They also report feeling numbness and unresponsiveness in their limbs and sometimes the sensation of pinpricks. Many have reported that they benefit from sports and light physical activity such as walking, yoga, and Pilates. Lymphoedema, which is a frequent consequence in women with breast cancer, also brings various problems such as swelling of the arm and pain, and physiotherapy is also required to allow the lymph to drain. Finally, many women report gynaecological problems such as vaginal hypertrophy. The consequences of these characteristics on daily activities include, above all, difficulties in household management. Pain also causes trouble in getting out of bed or off the couch and problems at work. Pain is often accompanied by physical tiredness.

*“I do things as if I am already tired, I should be more agile in my movements but I do them with tiredness.”*

*(C., 37 years old)*

Interestingly, almost the entire sample reported the feeling of “feeling old before my time”, which causes discomfort, anger, and nervousness. Many women cannot accept the presence of chronic and widespread pain at a young age and worry about the future; the thought of having to limit activities, such as going out with family and friends, leads to feelings of despondency.

*“Sometimes I can’t do daily activities, I get tired and feel 80 years old.”*

*(A., 43 years old)*

Subtheme 2: Resilience

It is possible for an individual to express good adaptive capacities in highly problematic situations from a very early age, bringing out latent personal resources that were not previously evident. The combination of these resources has been termed “resilience”, which is the ability to react to a high-stress condition or a traumatic event by continuing to grow effectively and come out stronger [26].

*“I’m not a fearful person, I face everything from the first moment, I’m sure I have a strength... my husband calls it alien strength (...) I had to roll up my sleeves, I never got discouraged.”*

*(A., 43 years old)*

*“I don’t stop, I get up, I put on make-up and I fix myself up, in fact the sicker I am the more I put on make-up (...) I can’t stop, I mustn’t stop, even if sometimes it’s hard.”*

*(M., 40 years old)*

Many of the participants reported that they had revolutionised their lives as a result of cancer and its aftermath; they found adaptability to adversity that they thought they did not have. Most of the sample reacted to the pain by joining a gym, walking, and practicing Pilates, while others, as reported in the record, defied the suffering by taking extra care, proving that nothing can bring them down. Interestingly, this aspect that emerged from the interviews is confirmed by numerous publications, which have led to cancer being considered not only as a traumatic event, a source of negative consequences, but also as a catalyst for growth [27].

#### 3.2.2. Theme 2: Relational Well-Being

The women interviewed expressed a very common feeling of not feeling understood, which also led to a willingness not to talk about their problems related to the disease. Chronic pain sometimes discourages participants from taking part in shared family activities involving physical exertion and often interferes with intimate relationships with husbands, especially due to hormone therapy. Not all women find benefit in opening up to their loved ones, but membership of cancer support groups was found to be a key support for many of them.

*“Chronic pain interferes with the relationship with family and friends, yes, because there are days when I am not particularly well, even morally perhaps, and they don’t understand it. I’ve been told “OK, but now you have breast implants” or “come on, you haven’t had chemo, you haven’t lost your hair” but what does that mean? You don’t say these things.”*

*(D., 48 years old)*

Subtheme 1: The Feeling of not feeling understood

The feeling of not feeling understood by others felt by the participants regarding their condition is the aspect that comes up more frequently than any other. Almost all women report that they do not feel understood by family members or friends, as it would be impossible for them to fully understand how they feel. This leads the participants to not find a confidant figure in their loved ones but rather to turn in on themselves.

*“I vent more with my husband; he is my outlet. But many times, I avoid involving others because I don’t really feel understood. They know I take the tablets (hormone pill); they know there are side effects but they don’t really understand when you are in that situation what it feels like.”*

*(C., 37 years old)*

The importance of the role played by associations is mainly linked to the possibility of bringing together women with the same experiences. The opportunity to discuss concerns, the consequences of surgery and treatment and, particularly in this case, long-term pain, allows each of them to feel truly understood, creating a strong support network.

*“The relationship created with all the members of the association has helped me a lot, there is a rare feeling between us (...) it is as if we had known each other all our lives, we share experiences (...) we are in tune, on the same track. The relationship and the dialogue with them are very useful, spontaneous, simple and natural, it also helps with pain management.”*

*(A., 43 years old)*

Subtheme 2: Willingness to protect loved ones

In almost all the interviews analysed, it emerged that there was a desire not to involve not only children but also husbands and parents in the difficulties related to the illness, so as not to make them suffer. Many women have decided to take full responsibility for their own suffering and pain, believing that there is no point in venting to their loved ones as this would only lead to unnecessary worry.

*“I was trying to be a normal mum, I didn’t want to burden the children with the pain, I was trying to absorb it all myself and not pour it on others.”*

*(M., 49 years old)*

#### 3.2.3. Theme 3: Perception and Role of the Psychologist

The sample in question is divided into exactly two halves: fifteen women received psychological support and the other fifteen chose not to. Concerning family members, only those of 2 women (6.66%) felt they needed psychological assistance, while the remaining 28 women (93.33%) did not. However, during the interviews, many participants stated that psychological support for family members, especially the husband, could not only help them in their treatment, but could also help their family members to react differently to the inevitable difficulties of the disease.

*“My family would need it very much. My husband experienced my disease in a special way, I was the one who had to give him a boost because he started throwing himself on the sofa and crying without doing anything else for a long time. He has major depression, there’s nothing to do.”*

*(G., 63 years old)*

Subtheme 1: Improvements perceived by users

Almost the entire sample who had received psychological support considered it to be fundamental throughout their cancer journey. Even the perception of pain was modified thanks to the figure of the psychologist. Many women reported that they had gone through a difficult process of accepting the disease, and thanks to this result, their perception of chronic pain also changed completely, being accepted as an inevitable consequence that one should not fight against but try to reduce and live with.

*“The acceptance of what I had, of what I had to do, of treatments, was difficult for me and only with the help of the therapy that we do as a group I had very good results (...) in these five years I can say that everything has changed and everything has improved, the pain cannot be taken away by the therapy but it can give me a different light and make me face it differently, I am certainly not facing it as I did before the psychological approach.”*

*(A., 49 years old)*

Psychological support was considered helpful by some women in not feeling alone. Some women reported needing psychological support due to the high anxiety they felt following the diagnosis and the total lack of physical and psychological strength following chemotherapy. Only two women in the sample reported that they did not continue the psychological course mainly due to lack of time, the restrictions caused by the pandemic, and because they felt that, after all, they had not benefited from it.

Subtheme 2: Reasons for not making use of the service

The remaining half of the participants reported that they had not used psychological support, each presenting their reasons. Most women felt that they did not need it; for many, their strength was such that they were able to keep the situation under control without external help, and thanks to the support provided by the family.

*“I have never considered psychological support because I believe I have found my balance.”*

*(C., 40 years old)*

Some of the participants, on the other hand, claimed that they did not have enough time, mainly because of the distance of the service from their homes and because of the children they had to take care of. The pandemic situation has made things worse because of the restrictions, the fear of contracting the virus and therefore of leaving home, and the fact that many services, psychological and not, have been interrupted. Other women stated that, at the time of their diagnosis, they had not been aware of the possibility of free psychological support in the facility where they were being treated and that, had they known, they would certainly have requested it.

## 4. Discussion

A breast cancer diagnosis constitutes a potentially traumatic event and a chronic stressor for women who experience it [14,17]. Depression, anxiety, and pain are very common and may heavily worsen quality of life [15,16,18]. With regard to the impact of chronic pain on daily activities and psychological well-being, it has been confirmed that it co-occurs with psychological distress and has a strong negative impact on quality of life, particularly with high levels of perceived pain severity [4]. In the current study, we investigated through a qualitative methodology the patient’s experience of chronic pain and how they perceived the role of psychologists. 

Strong feelings of anger and nervousness about the condition and difficulty in getting to sleep [4] were reported from the test sample, although this is greatly influenced by the hormone therapy that many follow. What is not in accordance with the literature is loss of appetite and any diagnosis of depressive or anxiety disorder. Although some typical symptoms of these disorders have been sporadically present, the conditions do not qualify as pathological. Lack of energy has been identified as the main cause of discomfort and as a motivation to avoid some of the most demanding activities [9]. Finally, a close relationship was shown between cognitive and emotional aspects and the perception of pain [28]. 

However, it is relational well-being that is most affected by the incidence of chronic pain. As it seems from the results, the overwhelming majority of women have a strong feeling of not feeling understood, which is absolutely in line with scientific findings [11]. Participants who do not feel understood prefer to avoid venting and opening up to their family and friends, as they do not find a reference point in them. What emerges as an innovative aspect, however, is the fundamental importance of voluntary associations, not so much as moral support at every stage of the cancer pathway, which is now well-known, but as support for the management of chronic pain. Arguably, there was also a tendency to avoid strenuous activities such as long walks or family outings for fear of not being able to cope with the pain, as depicted by the scientific findings [9]. However, contrary to expectations, many women claimed that they always try hard not to give up many things, instead deciding to challenge and cope with the pain. As our results reveal, many women showed resilience when struggling with persistent pain. Whatever the cause, research in clinical psychology mainly focusing on the negative consequences of cancer (i.e., psychological and physical symptoms) may be shortsighted. Perhaps not surprisingly then, research reveals that resilience is very common among individuals struggling with traumatic experiences [29], while post-traumatic growth may coexist with emotional distress, including depression and anxiety [30].

A final aspect that seems not to have been explored by scientific research so far are issues relating to the intimate sphere. Most women claim that their relationship with their husband has been challenged, and sometimes totally undermined, by the physical pain of hormone therapy.

Finally, both the participants who chose to use psychological support and those who did not, expressed, in the majority, absolutely positive opinions about the usefulness of the support. However, it is also true that none of them requested psychological support specifically for chronic pain and that this topic was treated sporadically during the meetings. Somewhat surprisingly, it is therefore evident that no specific therapy typical of psycho-oncology was implemented [19]. The fact that psychosocial factors can be determinant in pain management corroborates the view that the psychological interventions ought to be integrated into a multidisciplinary approach for women with breast cancer [31,32]. Thus, reducing mistaken beliefs about pain should be a primary target for specialists within the field, together with a careful consideration of psychological components. 

That said, some women reported that they had made a journey of acceptance of the disease and its consequences, and the type of work performed is still the basis of acceptance and commitment therapy [18]. The cognitive changes in the way of seeing and perceiving pain following psychological support also demonstrate the validity of the theories underlying cognitive–behavioural therapy [28]. Likewise, cancer narration supporting sensemaking processes may bolster adaptation to the disease [32]. In addition to helping us to better grasp how breast cancer impacts patients, knowledge of the cancer narration would have many other practical implications for clinical practice. 

The results of this qualitative study may be indeed useful for a deeper understanding of the relationships between chronic pain, quality of life, relational well-being, and the role of the psychologist. The experiences of the participants interviewed seem to provide direct insight into the difficulties and suffering associated with both illness and pain and were the fundamental core of the research.

From their words, some points of interest emerged that could be explored further in the future, such as the need for advice from a sexologist for the many problems arising from breast cancer but also from chronic pain and the hormonal pill. Another element that should be taken into account is the need to promote and foster the possibility of having inpatient psychological support. Many women are not aware of some free services in hospitals, which greatly influences their choice not to seek support. Others reported feeling abandoned in the aftermath of recovery, characterised only by regular check-ups and no interest in the pain and consequences of treatment. Some participants who took part in this study also expressed the need to improve the quality and consistency of psychological support in the outpatient setting, which they considered to be the most difficult time to deal with.

Although our findings add evidence in the understanding of the consequences of chronic pain among women with breast cancer, there also some limitations that future research should address. First, the cross-sectional design did not allow us to deduce changes over time in the three themes. Second, the heterogeneity of certain characteristics (i.e., the stage of disease) may have influenced the results and thereby may not fully generalizable to other patients with breast cancer. Third, other individual variables not taken into consideration could have impinged on the results. Indeed, personality factors and perceived social support may have an effect on the narratives of the patients. 

## 5. Conclusions

In sum, the findings of this study suggest that more attention needs to be paid to the quality of life of cancer patients, during and after the illness, and that more space should be given to the role of the psychologist in managing the consequences of their condition. Chronic and persistent pain should be addressed in all its physical, emotional, cognitive, social, and cultural aspects through the use of both medical and psychological therapies. The results that emerged should prompt reflection on the unacceptability of young women with their whole lives ahead of them complaining of these problems, without pain therapy that respects women’s overall needs. It is time to provide equal care and attention to both physical and psychological well-being because only in this way can we attempt to achieve the total promotion of quality of life.

## Figures and Tables

**Table 1:** Characteristics of participants.

Characteristics of Participants	Total (n = 29)
**Age:** mean years (sd)	51.06 (9.51)
**Currently being treated:**	
yes (%)	20 (68.96%)
no (%)	9 (31.03%)
**Type of treatment:**	
surgery	27
chemotherapy	23
oral chemotherapy	2
hormone therapy	25
radiotherapy	17
biological therapy	1
immunotherapy	1

**Table 2:** Emerged themes and subthemes.

Theme	Subtheme
Quality of life and psychological well-being	Common features of chronic pain and consequences Resilience
Relational well-being	Not feeling understood Willingness to protect loved ones
Perception and role of the psychologist	Improvements perceived by users Reasons for not making use of the service

## Data Availability

The data presented in this study are available on request from the corresponding authors.
